# Development of a triculture based system for improved benefit/risk assessment in pharmacology and human food

**DOI:** 10.1186/1753-6561-5-S8-P67

**Published:** 2011-11-22

**Authors:** Alexandra Bazes, Géraldine Nollevaux, Régis Coco, Aurélie Joly, Thérèse Sergent, Yves-Jacques Schneider

**Affiliations:** 1 Biochimie cellulaire, nutritionnelle & toxicologique, Institut des Sciences de la Vie & UCLouvain, Croix du Sud 5/3, 1348 Louvain-la-Neuve, Belgium

## 

Caco-2 cells are widely used for both mechanistic studies in molecular cell biology as well as for studies aiming at estimating, *in vitro*, the bioavailability of drug candidates, xenobiotics, food compounds …. This results largely from the spontaneous differentiation of this human colon adenocarcinoma line into enterocytes-like cells in classical culture conditions, as well as from the use of bicameral culture inserts including porosity calibrated filters.

Although Caco-2 cells represent the current “golden standard” of *in vitro* models of the human intestinal barrier, there is a trend to develop new systems mimicking more closely the intestinal epithelium for pharmaceutical and toxicological studies. In particular, goblet cells may bring a main constituent of the intestinal barrier as secreting mucus and contribute to the permeability met *in vivo*. In addition, gut associated M cells offer a putative way for oral delivery of nanoencapsulated therapeutic peptides and mucosal vaccines.

## Material and methods

The triculture model was adapted by combining the *in vitro* model of the human follicle-associated epithelium (including M-like cells) according to the protocol of des Rieux *et al.* (2007) and the co-culture of Caco-2 and HT29-5M1 cells from Nollevaux *et al*. (2006). Briefly, Caco-2 cells with or without HT29-5M1 cells were seeded on 12-wells Transwell inserts (3.0 μm pore diameter, Costar, Elscolab, Kruibeke, BE) and cultivated for 3 days in DMEM supplemented with 10 % foetal calf serum (FCS). Some inserts were then inverted and a piece of silicon rubber (Labo-Modern, Queveaucamps, BE) placed around the basolateral side. Then, inserts were transferred into a Petri dish pre-filled with culture medium and maintained for 10 days with the basolateral medium refreshed every 2 days. Raji-B cells, suspended in DMEM + 10% FCS, were added to the basolateral compartment of the inserts. The tricultures were maintained for 5 days. Co-cultures were cultured under the same conditions, but without the addition of Raji-B cells or HT29-5M1 cells.

Cell monolayer integrity, both in mono-, co- and tri-cultures, was controlled by measurement of the transepithelial electrical resistance (TEER, Millipore Millicell^®^ ERS, Billerica, MA) and by evaluation of cell permeability to Lucifer Yellow (LY; 457 Da, Sigma-Aldrich, St-Louis, MO).

After integrity experiments, cells were washed in PBS, fixed with acetone and permeabilised with 1% (v/v) Triton X-100. Cells were then washed with PBS, blocked with 1% BSA and washed again with PBS, and then incubated with mouse anti-MUC5AC at 1:100 dilution, rinsed in PBS and incubated with Alexa Fluor 488 goat anti-mouse 10µg/ml and Rhodamine-Phalloidin 4U/ml (Invitrogen, Eugene, OR). Finally, cells were mounted with Ultracruz Mounting Medium (Santa Cruz Biotechnology, Santa Cruz, CA). The monolayers were viewed using a Zeiss LSM 710 confocal laser scanning microscope (Carl Zeiss MicroImaging GmbH, Jena, DE).

## Results and discussion

To validate the triculture system as an intestinal barrier model, transport studies were used to determine the culture integrity and the permeability capacity with TEER measurement and assessment of the passage of LY. Furthermore, a morphological analysis was initiated to evaluate the proportion of the M cells, goblet cells and enterocytes and their implication in the intestinal epithelium model.

### Cell monolayer integrity and permeability properties

A decrease of TEER was observed in the different co-culture systems. This should, most probably, be correlated to an increase of the barrier permeability, upon addition of HT29-MTX or/and Raji cells in the system (Table 1).

**Table 1:** *In vitro* models characterisation: Cell monolayer integrity was determined by LY transport. LY was incubated with cell monolayers for 180 min at 37°C (n=3) and it was quantified by fluorimetry. The results were expressed as % of total fluorescent.

	Caco-2	HT29-MTX	Caco-2/HT29-MTX	Caco-2/Raji	Caco-2/HT29-MTX/Raji
TEER (Ω.cm^2^)	283±70	122±31	122±19	88±27	60±17
Lucifer Yellow transport (% fluorescence)	0,40±0,02	6,42±0,09	3,25±0,06	7,16±0,23	5,07±0,22

The effect of the addition of goblet-like cells or of the conversion of Caco-2 cells into M-like cells suggests a role of these cells in the ability of the intestinal cells to increase their absorbtion of hydrophilic molecules, as observed by the increase of the permeability of LY in the co- and tri-culture systems in comparison with the control.

The contribution of the different cell types in the triculture system further allows the integration of mechanisms such as transcytosis (M cells), the presence of mucus (goblet-like cells), and the effectiveness of the tight junctions in the culture system, as compared to a standard Caco-2 model.

### Localisation of the intestinal cell types

**Figure 1:**
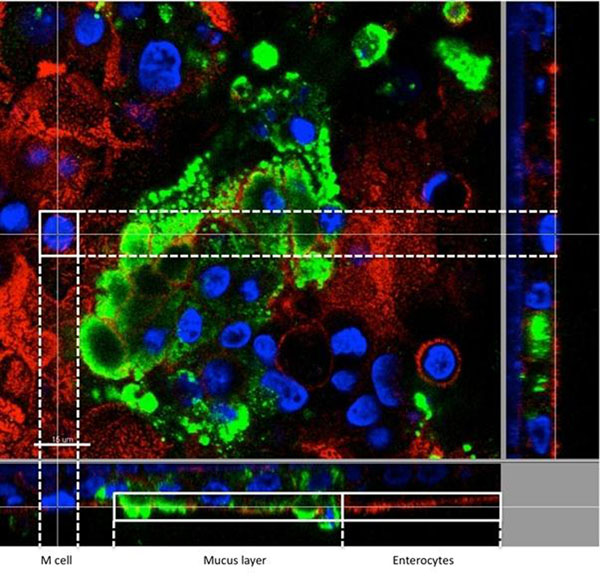
**Immunofluorescent localization of different cell types.** The nuclei of cells were stained in blue with the mounting medium. Enterocyte actin was stained with rhodamine-phalloidin (red) and goblet-like cells were MUC5AC-labelled (green). The M-like cells were detected by their lack of microvilli.

The intestinal cell types were detected by fluorescent labelling and confocal analysis (Figure 1). Actin (red) and MUC5AC (green) were restricted respectively on enterocyte and goblet-like cells. The M-like cells appeared within the enterocyte layer and were identified by their lack of microvilli (actin) at their apical surface. The mucus produced by goblet-like cells was on the surface (MUC5AC). In the triculture system, there was close contact between HT29-MTX, Raji and Caco-2 cells but the goblet-like cells have tendency to cluster together.

## Conclusion, perspectives

These results clearly indicate that the cocultivation of 3 different cell types allows to reconstitute a cell culture system that should better mimic the intestinal barrier, specially to investigate its interaction with nanoparticles. This should facilitate a better evaluation of pharmacological or toxicological properties of these materials in food and drugs.

We are now developing additional co-culture systems involving f.i. RAW264.7 macrophages to further determine the interactions involved in inflammatory processes; HepG2 cells to estimate the intestinal and hepatic effects in presystemic biotransformations.
